# Prevalence of Medical Disorders During Pregnancy in India: A Comprehensive Observational Study to Assess the Prevalence of Hypertension, Diabetes, and Thyroid Disorders During Pregnancy in Indian Women

**DOI:** 10.7759/cureus.86441

**Published:** 2025-06-20

**Authors:** Jalagam Revathi, Meena TS, Pavithra M

**Affiliations:** 1 Obstetrics and Gynecology, Sree Balaji Medical College and Hospital, Chennai, IND

**Keywords:** diabetes mellitus, hypertension, hypothyroidism, non-communicable disease (ncd), pregnancy

## Abstract

Background

Pregnancy is a critical period where maternal non-communicable diseases (NCDs) such as hypertensive disorders, gestational diabetes mellitus (GDM), and thyroid dysfunction significantly affect maternal and fetal outcomes. This study aimed to determine the prevalence and associated factors of these conditions among pregnant women in India.

Methods

A cross-sectional study was conducted among 300 pregnant women attending antenatal clinics at a tertiary care hospital. The participants were enrolled irrespective of gestational age. Known cases of pre-existing hypertension, diabetes, and thyroid disorders were excluded. Data were collected via structured questionnaires and clinical records, capturing demographics, obstetric history, lifestyle, diagnostic values, and the awareness of risk factors. Conditions were diagnosed based on standardized criteria (Diabetes in Pregnancy Study Group India {DIPSI} for GDM, blood pressure {BP} of ≥140/90 mmHg for hypertension, and trimester-specific thyroid-stimulating hormone {TSH} levels for thyroid dysfunction).

Results

The prevalence of GDM, hypertension, and thyroid dysfunction was 60 (20%), 48 (16%), and 36 (12%), respectively. GDM and hypertension were significantly more common in urban participants (p<0.05), and both were associated with higher body mass index (BMI). Thyroid dysfunction was frequently diagnosed in the first trimester (22, 61.1%). A significant upward trend in all three disorders was observed with increasing BMI. The awareness of risk factors was higher among educated women, correlating with earlier diagnosis.

Conclusion

This study highlights a substantial burden of NCDs among pregnant women, especially GDM and hypertension. Urbanization, obesity, and maternal age were key contributing factors. While structured screening and early antenatal registration are existing mandates, our findings underscore the need for targeted and scalable interventions beyond current protocols.

## Introduction

Pregnancy represents a remarkable physiological journey characterized by complex hormonal, metabolic, and immunological adaptations that support fetal development. However, it is also a period during which underlying health conditions may manifest or previously controlled disorders may become exacerbated. Among the most prevalent and clinically significant medical complications encountered during pregnancy are hypertensive disorders, gestational diabetes mellitus (GDM), and thyroid dysfunction. These conditions not only are common but also carry profound implications for both maternal and fetal outcomes, necessitating timely diagnosis and effective management to mitigate adverse effects [1].

If not appropriately addressed, these disorders can result in a spectrum of complications ranging from preterm delivery, low birth weight, and intrauterine growth restriction to stillbirth and a higher likelihood of cesarean deliveries [2,3]. Consequently, they represent a considerable burden on antenatal care (ANC) systems, particularly in resource-limited settings where early detection and consistent follow-up may be challenging. Their increasing incidence globally, and particularly in low- and middle-income countries such as India, underscores the urgency for targeted public health interventions and evidence-based clinical guidelines.

In the Indian context, the rising prevalence of non-communicable diseases (NCDs) among women of reproductive age has emerged as a significant public health concern. Factors such as rapid urbanization, sedentary lifestyles, changing dietary patterns, and increasing maternal age have contributed to a greater incidence of conditions such as hypertension, diabetes, and thyroid dysfunction during pregnancy [4]. As more Indian women conceive at older ages and amidst higher baseline metabolic risks, antenatal care providers are witnessing an escalating burden of these disorders. These trends have sparked growing attention among clinicians, researchers, and policymakers regarding the need for systematic screening and management protocols during pregnancy [5].

Hypertensive disorders in pregnancy, including gestational hypertension, preeclampsia, and eclampsia, rank among the leading causes of maternal morbidity and mortality in India [6]. These conditions are known to contribute to life-threatening complications such as stroke; hemolysis, elevated liver enzymes, and low platelet count (HELLP) syndrome; and multi-organ dysfunction in mothers, alongside serious fetal complications such as placental abruption, intrauterine fetal demise, and growth restriction [7]. Indian studies have reported prevalence rates ranging from 5% to 8%, although these figures vary widely depending on region, population characteristics, access to healthcare, and levels of maternal health awareness [8,9].

Similarly, GDM has emerged as a growing concern in Indian antenatal care. Prevalence estimates vary between 10% and 20%, depending on diagnostic criteria and geographic distribution [10]. Risk factors commonly associated with GDM in India include obesity, family history of diabetes, sedentary behavior, and advanced maternal age. GDM not only poses immediate risks such as macrosomia, neonatal hypoglycemia, and preeclampsia but also has long-term consequences. Both mothers and offspring affected by GDM have an elevated risk of developing type 2 diabetes mellitus later in life, making GDM a crucial focus for lifespan health interventions [11].

In addition, thyroid dysfunction, particularly hypothyroidism, is frequently observed during pregnancy and often remains undiagnosed due to its nonspecific symptoms, especially in the first trimester. In rural and under-resourced regions, iodine deficiency continues to contribute significantly to thyroid abnormalities. Studies estimate that approximately 10%-13% of pregnant Indian women experience thyroid dysfunction during gestation [12,13]. If left untreated, these conditions can lead to severe consequences, including miscarriage, preterm birth, impaired fetal neurodevelopment, and even stillbirth.

Despite the evident clinical relevance of these disorders, nationally representative data on their combined burden during pregnancy in India are lacking. Most existing literature consists of regional studies or focuses on a single condition, making it difficult to formulate a comprehensive understanding of epidemiological trends across the country. As a result, the development of uniform screening guidelines and targeted interventions remains a challenge. This study, therefore, aims to evaluate the prevalence of hypertensive disorders, GDM, and thyroid dysfunction among pregnant women in diverse demographic and socioeconomic settings across India. By identifying prevalence patterns, associated risk factors, and gaps in antenatal care, this research seeks to generate evidence-based insights that can inform public health policy and enhance maternal-fetal health outcomes in India.

## Materials and methods

Study design

This study was designed as a questionnaire-based, cross-sectional observational study with the primary objective of assessing the prevalence of three major medical disorders during pregnancy, hypertensive disorders, gestational diabetes mellitus (GDM), and thyroid dysfunction, among Indian women. The study was conducted over a three-month period, from February 2025 to May 2025. The cross-sectional nature of the design enabled a snapshot analysis of the occurrence of these conditions within a defined population at a particular point in time, which is especially useful for understanding disease burden and guiding antenatal care strategies.

Study setting

The study was carried out in the Department of Obstetrics and Gynecology at Chennai in a tertiary care teaching hospital. The antenatal care outpatient department (ANC OPD) served as the primary setting for participant recruitment. This center caters to a diverse patient population, encompassing individuals from both urban and rural settings, which adds depth to the demographic and socioeconomic representation of the study sample.

Study population

The study population comprised pregnant women aged 18 years and above, at any stage of gestation, who attended the antenatal clinic during the study period. A purposive sampling method was employed to ensure that the participants who were either at risk for, or being evaluated for, hypertension, GDM, or thyroid dysfunction were adequately represented. Inclusion in the study required the provision of informed consent, and women who had been previously diagnosed with chronic hypertension, pre-existing diabetes mellitus, or thyroid disorders prior to pregnancy were excluded to maintain the focus on conditions arising during gestation.

The primary objective of this study was to determine the prevalence of hypertensive disorders, diabetes, and thyroid dysfunction during pregnancy in an Indian population. The secondary objectives included identifying the gestational age at which these conditions were first diagnosed; analyzing variations in prevalence based on residence (urban versus rural), socioeconomic status, parity, and maternal age; and assessing awareness levels regarding risk factors for GDM and gestational hypertension among the participants.

Sample size

The sample size was calculated using a 95% confidence interval (1.96), with the estimated prevalence (19%) from previous literature, and the margin of error was set at 0.05 using OpenEpi 3.1 (Dean AG, Sullivan KM, Soe MM. OpenEpi: Open Source Epidemiologic Statistics for Public Health, Version. www.OpenEpi.com, updated 2013/04/06) [13]. A minimum of 238 participants was required; however, to enhance the robustness of the analysis and accommodate subgroup comparisons, the final target sample was increased to 300 participants.

Data collection tool and procedure

Data for the study were collected using a structured questionnaire (mentioned in the Appendices) administered by data collectors, which ensured both accessibility and efficiency in data gathering. The participants were selected using a purposive sampling technique. Women with pre-existing diagnoses of chronic hypertension, diabetes mellitus, or thyroid disorders prior to pregnancy were excluded to specifically assess the prevalence of conditions developing during gestation. The questionnaire was carefully designed to capture comprehensive information relevant to the study’s objectives and was divided into five major sections. The first section focused on demographic characteristics, including variables such as age, body mass index (BMI), educational attainment, household income, and place of residence (urban or rural). The second section documented the obstetric history of each participant, covering current gestational age, parity, and the nature of the pregnancy (singleton or multiple). The third section addressed lifestyle and behavioral factors, specifically dietary habits and levels of physical activity, which are known to influence the development of metabolic disorders during pregnancy. The fourth section captured the family history of chronic conditions such as hypertension, diabetes, and thyroid disorders, offering insight into hereditary risk factors. The final section collected clinical and diagnostic data, including blood pressure (BP) measurements, oral glucose tolerance test (OGTT) results, and thyroid-stimulating hormone (TSH) levels.

Prior to the initiation of data collection, all participants were thoroughly briefed about the study’s purpose and procedures. Informed consent was obtained in accordance with ethical guidelines, ensuring that participation was voluntary and fully understood. Trained investigators conducted in-person interviews with each participant in the local language (Tamil), and where applicable, information was supplemented by reviewing clinical records and laboratory investigation reports to ensure accuracy and completeness.

Standard diagnostic criteria were employed to classify the medical conditions under investigation. Hypertensive disorders were defined as the presence of blood pressure readings equal to or exceeding 140/90 mmHg after 20 weeks of gestation, regardless of the presence of proteinuria or signs of organ dysfunction. For the diagnosis of GDM, the Diabetes in Pregnancy Study Group India (DIPSI) criteria were used, where a two-hour plasma glucose level of 140 mg/dL or higher following the administration of a 75 g oral glucose load was considered diagnostic. Thyroid dysfunction was assessed using trimester-specific TSH reference ranges, with a TSH level above 2.5 mIU/L during the first trimester typically considered abnormal. These clinical benchmarks ensured that all conditions were identified based on established guidelines, enhancing the study’s internal validity and clinical relevance.

Ethical considerations

The study protocol was approved by the Institutional Human Ethics Committee of Sree Balaji Medical College and Hospital (approval number: 002/SBMCH/IHEC/2025/2399), ensuring adherence to ethical standards in human research. Participation was entirely voluntary, and no financial or material incentives were offered. Confidentiality and privacy were strictly maintained throughout the study, with no personally identifiable information recorded.

Data analysis

Data were entered into a secured database and analyzed using Stata version 17.0 (StataCorp LLC, College Station, TX). Descriptive statistics were used to summarize demographic characteristics and prevalence rates. Chi-square/Fisher’s exact tests were employed to assess associations between categorical variables. Subgroup analyses were conducted to evaluate differences in disease prevalence by maternal age, residence (urban versus rural), socioeconomic status, and parity. A p-value of <0.05 was considered statistically significant.

## Results

Table 1 presents the demographic characteristics of the 300 pregnant women who participated in the study. The majority of participants were between 26 and 35 years of age, accounting for 140 women (46.7%). In terms of residence, a larger proportion of participants were from urban areas (180, 60%), while 120 women (40%) resided in rural settings. Regarding educational attainment, 100 participants (33.3%) had completed higher secondary education, and 30 (10%) had only primary education. When classified by body mass index (BMI), 150 participants (50%) had a normal BMI (<25), 100 (33.3%) were overweight (BMI: 25-30), and 50 (16.7%) were classified as obese (BMI: >30). These demographic variables provided a diverse sample that allowed for meaningful subgroup analysis in relation to the prevalence of medical disorders during pregnancy.

**Table 1:** Demographic characteristics of the study participants (N=300) *WHO classification of obesity [14] BMI: body mass index

Variable	Category	Frequency (n)	Percentage (%)
Age (Years)	18-25	120	40.0%
	26-35	140	46.7%
	>35	40	13.3%
Residence	Urban	180	60.0%
	Rural	120	40.0%
Education Level	Primary	30	10.0%
	Secondary	90	30.0%
	Higher Secondary	100	33.3%
	Graduate or Above	80	26.7%
BMI Category (kg/m^2^)*	Normal (<25)	150	50.0%
	Overweight (25-30)	100	33.3%
	Obese (>30)	50	16.7%

Table 2 summarizes the overall prevalence of three common medical disorders during pregnancy among the 300 study participants. Gestational diabetes mellitus (GDM) was the most prevalent condition, diagnosed in 60 women (20%), followed by hypertensive disorders, which were observed in 48 participants (16%). Thyroid dysfunction was reported in 36 women (12%), highlighting its significance as a frequently underdiagnosed condition during pregnancy. These findings reflect a substantial burden of non-communicable disorders among pregnant women and underscore the need for routine screening and timely management as part of comprehensive antenatal care.

**Table 2:** Prevalence of medical disorders during pregnancy among the study participants (n=300)

Medical Condition	Number of Cases	Prevalence (%)
Hypertension	48	16%
Gestational Diabetes	60	20%
Thyroid Dysfunction	36	12%

Table 3 presents the trimester-wise distribution of diagnoses for each medical condition. Hypertension was most frequently diagnosed in the second trimester (41.7%), followed by the third (33.3%) and first trimesters (25%). Gestational diabetes showed a similar pattern, with the majority of cases identified in the second trimester (56.7%), followed by the third (33.3%) and first (10%). In contrast, thyroid dysfunction was predominantly diagnosed in the first trimester (61.1%), with fewer cases detected in the second (27.8%) and third trimesters (11.1%). This distribution emphasizes the importance of early pregnancy screening, particularly for thyroid disorders, and midpregnancy evaluations for hypertension and GDM.

**Table 3:** Distribution of medical conditions by trimester of diagnosis among the study participants

Condition	First Trimester	Second Trimester	Third Trimester
Hypertension	12 (25%)	20 (41.7%)	16 (33.3%)
Gestational Diabetes	6 (10%)	34 (56.7%)	20 (33.3%)
Thyroid Dysfunction	22 (61.1%)	10 (27.8%)	4 (11.1%)

Table 4 represents the association of medical conditions with the sociodemographic parameters in detail. Hypertension during pregnancy showed significant associations with age, residence, education level, BMI, and trimester of diagnosis. It was more common among women aged above 35 years (10 cases, 25%), followed by those aged 26-35 years (28 cases, 20%), and least among the 18-25 age group (10 cases, 8.3%) (p=0.01). Urban residents had a higher prevalence (36 cases, 20%) compared to rural women (12 cases, 10%) (p=0.02). Educational level was inversely related to hypertension: women with primary education had the highest prevalence (10 cases, 33.3%) compared to secondary (18 cases, 20%), higher secondary (14 cases, 14%), and graduate level (six cases, 7.5%) (p=0.001). BMI was strongly associated: 12 cases (8%) were found in women with normal BMI, 22 cases (22%) in overweight, and 14 cases (35%) in obese participants (p=0.001). Most cases were diagnosed in the second trimester (20 cases, 41.7%), followed by the third (16 cases, 33.3%) and first trimester (12 cases, 25%) (p=0.001).

**Table 4:** Association between various sociodemographic variables and medical disorders during pregnancy *P-value of <0.05 considered statistically significant BMI: body mass index

Medical Condition	Associated Variable	Category	n (%)	Chi-square (χ²)	P-value
Hypertension	Age (Years)	18-25	10 (20.8%)	9.21	0.01*
		26-35	28 (58.3%)		
		>35	10 (20.8%)		
	Residence	Urban	36 (75%)	5.17	0.02*
		Rural	12 (25%)		
	Education Level	Primary	10 (20.8%)	14.28	0.001*
		Secondary	18 (37.5%)		
		Higher Secondary	14 (29.2%)		
		Graduate or Above	6 (12.5%)		
	BMI (kg/m^2^)	Normal (<25)	12 (25%)	14.38	0.001*
		Overweight (25-30)	22 (45.8%)		
		Obese (>30)	14 (29.2%)		
	Trimester of Diagnosis	First Trimester	12 (25%)	13.21	0.001*
		Second Trimester	20 (41.7%)		
		Third Trimester	16 (33.3%)		
Gestational Diabetes	Age	18-25	12 (20%)	7.58	0.02*
		26-35	34 (56.7%)		
		>35	14 (23.3%)		
	Residence	Urban	44 (73.3%)	6.65	0.01*
		Rural	16 (26.7%)		
	Education Level	Primary	12 (20%)	16.91	0.001*
		Secondary	20 (33.3%)		
		Higher Secondary	16 (26.7%)		
		Graduate or Above	12 (20%)		
	BMI	Normal (<25)	14 (23.3%)	19.85	0.001*
		Overweight (25-30)	28 (46.7%)		
		Obese (>30)	18 (30%)		
	Trimester of Diagnosis	First Trimester	6 (10%)	12.54	0.002*
		Second Trimester	34 (56.7%)		
		Third Trimester	20 (33.3%)		
Thyroid Dysfunction	Age	18-25	8 (22.2%)	5.49	0.06
		26-35	20 (55.6%)		
		>35	8 (22.2%)		
	Residence	Urban	26 (72.2%)	3.08	0.08
		Rural	10 (27.8%)		
	Education Level	Primary	6 (16.7%)	8.23	0.04*
		Secondary	12 (33.3%)		
		Higher Secondary	10 (27.8%)		
		Graduate or Above	8 (22.2%)		
	BMI	Normal (<25)	10 (27.8%)	7.14	0.03*
		Overweight (25-30)	16 (44.4%)		
		Obese (>30)	10 (27.8%)		
	Trimester of Diagnosis	First Trimester	22 (61.1%)	18.89	<0.001*
		Second Trimester	10 (27.8%)		
		Third Trimester	4 (11.1%)		

Gestational diabetes mellitus (GDM) also showed significant associations with multiple variables. It was most prevalent among women aged above 35 years (14 cases, 35%), followed by those aged 26-35 years (34 cases, 24.3%), and least among those aged 18-25 years (12 cases, 10%) (p=0.02). Urban women had a higher prevalence (44 cases, 24.4%) than rural women (16 cases, 13.3%) (p=0.01). The highest frequency was seen among women with primary education (12 cases, 40%), compared to secondary (20 cases, 22.2%), higher secondary (16 cases, 16%), and graduates (12 cases, 15%) (p=0.001). Regarding BMI, GDM was found in 14 women with normal BMI (9.3%), 28 with overweight (28%), and 18 with obesity (45%) (p=0.001). Trimester-wise, the majority of diagnoses occurred in the second trimester (34 cases, 56.7%), followed by the third (20 cases, 33.3%) and first (six cases, 10%) (p=0.002).

Thyroid dysfunction was most frequently observed in women aged above 35 years (eight cases, 20%) and 26-35 years (20 cases, 14.3%), compared to 18-25 years (eight cases, 6.7%) (p=0.06). Urban women had a slightly higher rate (26 cases, 14.4%) than rural participants (10 cases, 8.3%) (p=0.08). Education was significantly associated with thyroid disorders, with the highest prevalence in those with primary education (six cases, 20%), followed by secondary (12 cases, 13.3%), higher secondary (10 cases, 10%), and graduates (eight cases, 10%) (p=0.04). The prevalence increased with BMI: 10 cases (6.7%) were seen in women with normal BMI, 16 (16%) in overweight, and 10 (25%) in obese women (p=0.03). Interestingly, thyroid dysfunction was most often diagnosed in the first trimester (22 cases, 61.1%), followed by the second (10 cases, 27.8%) and third trimesters (four cases, 11.1%) (p<0.001).

## Discussion

This study provides important insights into the prevalence of three major medical disorders, hypertension, GDM, and thyroid dysfunction, among pregnant Indian women. The findings reinforce existing literature while highlighting the growing burden of non-communicable diseases during pregnancy due to changing lifestyle patterns, rising maternal age, and urbanization.

The observed prevalence of hypertensive disorders in pregnancy was 16%, which aligns with earlier studies reporting a national prevalence between 5% and 15% [15,16]. Higher incidence was noted among urban participants, possibly due to greater stress, dietary sodium intake, and sedentary habits [17,18]. Most cases were diagnosed during the second trimester, consistent with routine antenatal screening schedules. Hypertensive disorders, particularly preeclampsia and eclampsia, are major causes of maternal and fetal morbidity and mortality in India and globally. Complications include preterm labor, placental abruption, intrauterine growth restriction, and stillbirth. The study supports current recommendations for early blood pressure monitoring, especially in women with risk factors such as obesity, advanced maternal age, and a family history of hypertension [19].

GDM was the most common condition in our study, affecting 20% of participants. This is consistent with prevalence rates in southern India, where GDM affects approximately 18%-22% of pregnant women, particularly in urban settings [20]. Most diagnoses occurred in the second trimester, coinciding with the standard glucose screening window. The analysis confirmed a strong association between GDM and obesity, with higher prevalence among overweight and obese women [1]. Additional risk factors included family history of diabetes, sedentary lifestyle, and advanced maternal age. Urban residence was also linked to higher GDM rates, reflecting modern dietary patterns and reduced physical activity [1]. GDM poses significant risks, including macrosomia, neonatal hypoglycemia, and long-term metabolic disorders in both mother and child. Nearly 40% of women with GDM in this study required pharmacological treatment, reinforcing the importance of timely diagnosis and personalized care [21].

Thyroid dysfunction was observed in 12% of participants, with hypothyroidism being the most prevalent. A majority of cases were diagnosed during the first trimester, which is ideal given the role of thyroid hormones in fetal brain development [22]. National data estimate thyroid dysfunction affects 10%-13% of Indian pregnant women, which supports our findings [23]. Rural participants showed slightly higher prevalence, possibly linked to iodine deficiency, delayed antenatal visits, and limited access to diagnostic facilities. Untreated thyroid dysfunction in pregnancy is associated with miscarriage, preterm birth, and impaired cognitive development in the fetus [24]. Yet, referral to endocrinologists was limited in our study population, pointing to gaps in the continuum of care.

This study identified significant correlations between sociodemographic factors and the prevalence of medical disorders. Urban women exhibited higher rates of hypertension and GDM, reflecting lifestyle-related risks, while rural women were more likely to be underdiagnosed [25]. Education and income levels were positively associated with awareness and early health-seeking behavior, consistent with findings from other Indian studies. These findings highlight the dual challenge of managing lifestyle-related risks in urban populations while addressing access and awareness issues in rural areas. Bridging this gap requires targeted antenatal care strategies and improved screening infrastructure across all demographic groups.

The trimester of diagnosis influenced outcomes. Thyroid dysfunction was often identified in the first trimester, while hypertension and GDM were more frequently detected during the second trimester. Early detection was associated with fewer maternal and neonatal complications, reinforcing the importance of structured screening protocols and timely registration in antenatal care [26]. Women diagnosed in the third trimester showed higher rates of complications such as preeclampsia, cesarean delivery, and neonatal distress. These findings support existing guidelines advocating first-trimester booking and comprehensive risk assessment [27].

This study highlights the need for integrating screening for non-communicable diseases into routine antenatal services. Early screening for hypertension, GDM, and thyroid disorders should become standard practice during the first antenatal visit. Strengthening referral systems and ensuring access to multidisciplinary care, including endocrinologists, nutritionists, and high-risk pregnancy specialists, is vital. Public awareness programs and community-based interventions can play a key role. Training frontline workers such as Accredited Social Health Activists (ASHAs) to educate women about risk factors and the importance of regular screening can enhance early diagnosis, especially in low-resource settings [28]. Mobile health initiatives and digital reminders could support this effort in both urban and rural areas.

Limitations

This study’s strengths include a diverse sample, standardized data collection via a structured digital questionnaire, and the inclusion of both rural and urban populations. However, being cross-sectional, it does not allow causal inferences. Self-reported lifestyle data may introduce recall bias, and some subclinical or undiagnosed cases might have been missed due to access limitations.

Implications

There is a need for longitudinal studies that track women from preconception through postpartum to better understand disease progression and long-term maternal and child health outcomes. Research into the genetic and environmental determinants of these disorders in Indian populations would also provide valuable insights. Interventional studies on community-based education, digital health tools for monitoring, and structured referral systems could further improve outcomes and healthcare delivery.

## Conclusions

This study highlights that hypertension, GDM, and thyroid dysfunction are significant health concerns among pregnant women in India. Their prevalence is notably associated with modifiable risk factors, including elevated BMI, lifestyle patterns, and disparities in access to healthcare services. The findings reflect a dual challenge: rising detection rates in urban populations due to improved screening and persistent underdiagnosis in rural settings due to limited awareness. These trends underscore the need for early and universal screening protocols, comprehensive patient education, and the implementation of integrated antenatal care models that address both clinical and sociodemographic determinants. Strengthening these areas can contribute meaningfully to improving maternal and fetal health outcomes. Further research should aim to develop and evaluate targeted interventions, along with long-term follow-up strategies, to effectively reduce the burden of these pregnancy-related medical disorders and their associated complications.

## Appendices

Table 5 shows the questionnaire administered by data collectors.

**Table 5:** Questionnaire performa Credits: TS Meena and M Pavithra BMI, body mass index; BP, blood pressure; TSH, thyroid-stimulating hormone; MNT, medical nutrition therapy

Section	Variable	Response Options/Entry
Demographics	Age (in Years)	☐ 18-25 ☐ 26-35 ☐ >35
	BMI	________ kg/m²
	Religion	☐ Hindu ☐ Islam ☐ Christian ☐ Others
	Education Level	☐ Primary School ☐ High School ☐ Higher Secondary ☐ Graduate ☐ Others
	Occupation	☐ Business ☐ Government Servant ☐ Private ☐Others
	Monthly Income	☐
	Trimester of Pregnancy	☐ ≤3 months ☐ 4-6 months ☐ ≥7 months
	Type of Family	☐ Nuclear ☐ Joint ☐ Extended
	Type of Pregnancy	☐ Planned ☐ Unplanned
	Conception Type	☐ Spontaneous ☐ After Infertility Treatment
	Duration of Marriage	☐ ≤5 years ☐ 6-10 years ☐ ≥11 years
	Residence	☐ Urban ☐ Rural
	Gravida	______
	Para	______
	Live Births	______
	Abortions	______
	Previous Information on Labor	☐ Yes ☐ No
Hypertensive Disorder in Pregnancy	Gestational Age at Diagnosis	_______ weeks
	BP at Diagnosis	______ / ______ mmHg
	Urine for Protein	☐ Nil ☐ Trace ☐ + ☐ ++ ☐ +++ ☐ ++++
	Treated With Oral Antihypertensives	☐ Yes ☐ No
	Required IV Medications/Hospital Admission	☐ Yes ☐ No
	Experienced Convulsions	☐ Yes ☐ No
Gestational Diabetes Mellitus (GDM)	Gestational Age at Diagnosis	_______ weeks
	Two-Hour Glucose Value	_______ mg/dL
	Treated With MNT	☐ Yes ☐ No
	Treated With Oral Hypoglycemics	☐ Yes ☐ No
	Required Insulin Therapy	☐ Yes ☐ No
Thyroid Dysfunction	Gestational Age at Diagnosis	_______ weeks
	TSH Level	_______ mIU/L
	Treated With Oral Medication	☐ Yes ☐ No
	Referred to Endocrinologist/Physician	☐ Yes ☐ No
